# Usefulness of the SF-36 Health Survey in screening for depressive and anxiety disorders in rheumatoid arthritis

**DOI:** 10.1186/s12891-016-1083-y

**Published:** 2016-05-23

**Authors:** Faith Matcham, Sam Norton, Sophia Steer, Matthew Hotopf

**Affiliations:** Department of Psychological Medicine, Institute of Psychiatry, Psychology and Neuroscience, King’s College London, London, UK; Psychology Department, Institute of Psychiatry, Psychology and Neuroscience, King’s College London, London, UK; Department of Rheumatology, King’s College London, London, UK

**Keywords:** Anxiety, Depression, SF-36, Rheumatoid Arthritis

## Abstract

**Background:**

This study aimed to assess the accuracy of the Short-Form Health Survey (SF-36) mental health subscale (MH) and mental component summary (MCS) scores in identifying the presence of probable major depressive or anxiety disorder in patients with rheumatoid arthritis.

**Methods:**

SF-36 data were collected in 100 hospital outpatients with rheumatoid arthritis. MH and MCS scores were compared against depression and anxiety data collected using validated measures as part of routine clinical practice. Sensitivity and specificity of the SF-36 were established using receiver operating characteristic (ROC) curve analysis, and area under the curve (AUC) compared the performance of the SF-36 components with the 9-item Patient Health Questionnaire (PHQ9) for depression and the 7-item Generalised Anxiety Disorder (GAD7) questionnaire for anxiety.

**Results:**

The MH with a threshold of ≤52 had sensitivity and specificity of 81.0 and 71.4 % respectively to detect anxiety, correctly classifying 73.5 % of patients with probable anxiety disorder. A threshold of ≤56 had sensitivity and specificity of 92.6 and 73.2 % respectively to detect depression, correctly classifying 78.6 % of patients, and the same threshold could also be used to detect either depression or anxiety with a sensitivity of 87.9 %, specificity of 76.9 % and accuracy of 80.6 %. The MCS with a threshold of ≤35 had sensitivity and specificity of 85.7 and 81.9 % respectively to detect anxiety, correctly classifying 82.8 % of patients with probable anxiety disorder. A threshold of ≤40 had sensitivity and specificity of 92.3 and 70.2 % respectively to detect depression, correctly classifying 76.3 % of patients. A threshold of ≤38 could be used to detect either depression or anxiety with a sensitivity of 87.5 %, specificity of 80.3 % and accuracy of 82.8 %.

**Conclusion:**

This analysis may increase the utility of a widely-used questionnaire. Overall, optimal use of the SF-36 for screening for mental disorder may be through using the MCS with a threshold of ≤38 to identify the presence of either depression or anxiety.

**Electronic supplementary material:**

The online version of this article (doi:10.1186/s12891-016-1083-y) contains supplementary material, which is available to authorized users.

## Background

Rheumatoid arthritis (RA) is a chronic, painful, progressive condition, which has a substantial impact on patients’ quality-of-life (QoL) [1]. The prevalence of depression in this condition is high, with a recent meta-analysis [2] revealing that an estimated 38.8 % of patients screen positive for probable major depressive disorder (pMDD) according to the 9-item Patient Health Questionnaire (PHQ9; [3]). Common mental disorders such as pMDD or probable generalised anxiety disorder (pGAD) can have implications for long-term health outcomes; depression and anxiety are associated with increased fatigue [4], impaired long-term disease activity and physical disability [5], and reduced treatment efficacy [6].

Despite its prevalence and importance, mental health is rarely measured either in rheumatological research or in clinical practice, reported as an outcome in less than 8 % of published research [7]. QoL is more frequently measured (in 19 % of studies), most often with the Short-Form Health Survey (SF-36 [8]) [7]. The SF-36 has been extensively validated as a measure of QoL in multiple populations and is the most widely used and evaluated QoL outcome measure [9]. The SF-36 consists of 8 domains, which assess physical function (PF), role physical (RP), bodily pain (BP), global health (GH), vitality (VI), social function (SF), role emotional (RE) and mental health (MH). Scores on these subscales can also be combined to create two higher-order summary scores: the physical component summary (PCS) and mental component summary (MCS). The PCS is calculated by positively weighting the 4 physical subscales (PF, RP, BP and GH), and by negatively weighting the psychological subscales (VI, SF, RE and MH). Conversely, the MCS is created by positively weighting the psychological subscales and negatively weighting the physical subscales.

There are several similarities between the SF-36 MH subscale and typical depression and anxiety screening questionnaire. Items relating to low mood (“Have you felt downhearted and low?”), tiredness (“Did you feel tired?”), nerves (“Have you been a very nervous person”) and restlessness (“Did you have a lot of energy”) are comparable to items such as “Feeling down, depressed or hopeless” (PHQ9 item 2), “Feeling tired or having little energy” (PHQ9 item 4), “Feeling nervous, anxious or on edge” (GAD7 item 1) and “Being so restless that it is hard to keep still” (GAD7 item 5). Additionally, the weighting of other QoL domains introduced when combining subscale scores for the MCS include other depression and anxiety symptoms, such as psychosomatic symptomatology and emotional interference with daily activities.

Validating the MH and MCS constructs within the SF-36 as screening tools for depression and anxiety may add extra utility to a questionnaire which is already frequently used for research purposes, and could also provide additional room for interrogation in clinical trial datasets which measure QoL but not mental health. The identification of useful thresholds can also have implications for implementing change in clinical practice. For example, patients attending general outpatient appointment at King’s College Hospital NHS Foundation Trust are required to complete the PHQ9 and GAD7 along with other patient reported outcomes, such as pain and fatigue visual analogue scales (VAS), and the Health Assessment Questionnaire (HAQ [10]) [11], on tablet devices while they wait for their appointment. The results of these assessments are made available immediately on their electronic health record, with advice for onward referral if required [11]. To date, the SF-36 MH and MCS scores have been validated as screening tools for depression and anxiety in an elderly population [12], however a similar validation process has yet to be performed in an RA sample.

We aimed to: 1) examine the relationships between MH and MCS SF-36 domains and depression, anxiety, and indicators of disease severity; 2) assess the accuracy of the MH and MCS SF-36 domains in identifying the presence of pMDD and pGAD, and describe the sensitivity and specificity of cut-off scores in the SF-36 MH and MCS in screening for psychological disorder; and 3) to recommend the most appropriate threshold with which to identify pMDD, pGAD, or presence of any psychological disorder (pMDD or pGAD).

## Method

### Setting

Data were collected using questionnaires administered to consecutive RA outpatients attending outpatient appointments at King’s College Hospital, an inner city hospital.

### Eligibility criteria

In order to be eligible to participate in this study several inclusion and exclusion criteria applied. Inclusion criteria were: 1) Having sufficient English to complete the questionnaire, or having at translator present to assist; 2) Able to give informed consent, i.e. no substantial learning disability or dementia. The following exclusion criteria were applied: 1) No clinic data collected within an appropriate timeframe (same day for psychological variables, ± 3 months for disease activity and disability measures); 2) Severe disability such as blindness or extreme frailty precluding the ability to answer independently.

### Procedure

Consecutive patients attending outpatient appointments were introduced by their clinicians to the researcher, who then confirmed eligibility and provided patients with the study information sheet. This information sheet explained the purpose of the research project, and confirmed that all responses would remain anonymous and be analysed in a confidential manner. Patients were also informed that they would be free to withdraw at any time.

Consenting participants were asked to provide their hospital number and asked to complete the SF-36. Patients were asked to either complete the SF-36 questionnaire before leaving the hospital, or were provided with a stamped addressed envelope to complete the questionnaire at home.

The SF-36 were combined with data collected routinely in clinic. This clinical data includes assessment of disease activity (DAS-28), and patient reported outcomes including: physical disability, pain and fatigue, as well as depression and anxiety. Every patient attending an outpatient appointment is asked to complete these patient-reported outcomes as part of their pre-appointment assessments. Patients can choose not to complete these assessments. To be eligible for inclusion in the current analysis, clinic data had to be collected on the same day as the SF-36, to ensure SF-36 data represented current mood and physical health. Hospital numbers were used to link questionnaire data with clinical data, and data were pseudonymised (with hospital numbers replaced with study identification numbers) by an independent database manager before release to the researcher. This procedure was approved by the Midlands National Research Ethics Service Committee (reference: 14/WM/0173).

### Recruitment

Data were collected between July 2014 and February 2015, on one day per week. This day was consistent across the recruitment period, as it had previously been identified as having several dedicated RA clinics running, therefore likely to yield the more eligible patients than other days of the week.

The target sample size was set at 100. This decision was based on the accuracy with which the area under the ROC curve (AUC) would be estimated. Specifically, for an AUC of 80 % or higher, width of the 95 % confidence interval would be no larger than +/− 8 %, which was deemed acceptable.

### Outcome measures

#### Depression

Depression was measured routinely in clinic using the 9-item Patient Health Questionnaire (PHQ9 [3]), which has been recommended by the National Institute for Health and Care Excellence (NICE) for use in adult patients with chronic physical health problems [13]. Probable major depressive disorder (pMDD) was defined as scoring “more than half the days” or “nearly every day” within the last two weeks on at least one of the first two items of the PHQ-9 (low mood and anhedonia), and on at least five out of all nine symptoms. This categorical algorithm for identifying pMDD with the PHQ9 has 83 % (95 % CI: 72–91 %) sensitivity and 90 % (95 % CI: 87–93 %) specificity when validated against the “gold standard” Structured Clinical Interview for DSM-IV for identifying pMDD, and has an overall accuracy of 89 % (95 % CI: 86–92 %) [14].

#### Anxiety

Anxiety was measured routinely in clinic using the 7-item Generalised Anxiety Disorder (GAD7) questionnaire. A score of 10 or more on the GAD7 has 89 % (95 % CI: 73–98 %) sensitivity and 82 % (95 % CI: 79–84 %) specificity for identifying the presence of probable generalised anxiety disorder (pGAD), when compared to psychiatric interviews [15].

#### Disease variables

Disease activity was quantified using the 28-joint disease activity score (DAS28). This DAS28 is recommended by all major RA guidelines and is considered to be the gold-standard indicator of disease activity [16]. The DAS28 takes into account subjective and objective markers of disease severity: erythrocyte sedimentation rate (ESR) and a clinician-recorded swollen joint count (SJC) provide objective indication of inflammation; patient-reported tender joint count (TJC) and a patient global assessment (PGA) provide subjective elements of disease activity. This are combined and weighted to form an overall DAS28 score, with higher scores indicating worsened disease activity. A scores of ≤2.6 would suggest the patient is in a state of remission; a score of 2.6–3.2 would suggest low disease activity; scores between 3.2 and 5.1 would indicate moderate disease activity; and scores of over 5.1 would suggest high disease activity [17].

The HAQ [10] is a patient-reported measure of physical disability. It contains questions relating to 8 domains of activities of daily living: dressing and grooming; rising; eating; walking; hygiene; reach; gripping and opening things; and daily activities. Scoring for each section ranged between 0 (“without any difficulty”) to 3 (“unable to do”), and total scores are on a scale of 0–3, with higher scores indicating higher levels of disability.

Pain and fatigue data were each collected via 0–100 visual analogue scales, with higher scores indicating increased levels of pain/fatigue.

#### SF-36

SF-36 subscales were calculated according to the SF-36 manual [18], resulting in 8 subscale scores: physical function (PF), role physical (RP), bodily pain (BP), general health (GH), vitality (VI), social functioning (SF), role emotional (RE), and mental health (MH). Physical component summary (PCS) and mental component (MCS) scores were calculated by norming subscale scores against population scores obtained from a normative UK dataset [19]. Normed subscale results were then weighted appropriately to calculate PCS and MCS totals, where a score of 50 represents the mean of the UK population (SD10).

### Statistical analyses

Linear regression analyses examined any differences in mental and physical health between patients who participated in the study and patients who did not, to assess for potential selection bias. Patients who did not have clinical data collected within the specified timeframe (same day for psychological variables, ± 3 months for disease activity and disability measures), had their next closest clinical data included in this analysis only, to test for significant differences in disease state between those recruited and the remaining clinical population. Pearson’s correlation analyses will assess the associations between PHQ9 depression scores, the SF-36 mental health subscale and mental component summary and: age; anxiety; disease duration; fatigue; pain; HAQ; DAS28 and its components TJC, SJC, ESR and PGA; CRP; BMI; the SF-36 subscales PF, RP, BP, GH, VI, RE, SF and PCS; and illness perceptions of consequence, timeline, personal control, treatment control, identity, concern, coherence and emotional representation.

Main analysis included sensitivity, specificity, predictive value and likelihood ratio assessment, along with ROC curve analysis to combine sensitivity and specificity. The AUC was used to compare the performance of the MH and MCS SF-36 components with PHQ9 to define depression and GAD7 to identify anxiety. Optimal thresholds for identifying depression with the MH and MCS were found through identifying the threshold which provided the highest level of sensitivity, with the least sacrifice in specificity. Positive and negative likelihood ratios described the accuracy of the SF-36 in detecting cases of pMDD and pGAD, with positive likelihood ratio thresholds of >10, 5–10, 2–5 and 1–2 indicating large, moderate, small (important), small (unimportant) changes in probability, and negative likelihood ratio thresholds of <0.1, 0.1–0.2, 0.2–0.5 and 0.5–1.0 indicating large, moderate, small (important), small (unimportant) changes in probability [20]. Analyses were conducted using STATA v11.

## Results

**Fig. 1:**
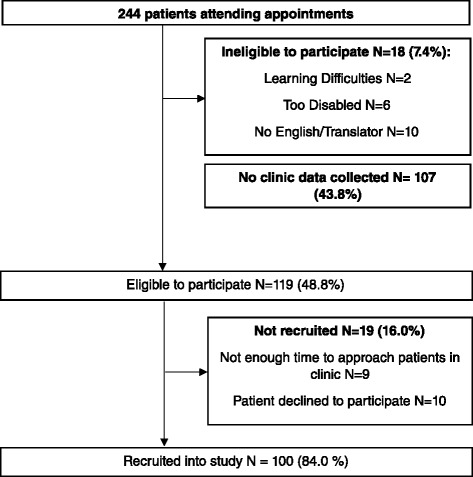
Flow chart of study recruitment

A total of 244 individual patients attended appointments throughout the recruitment period. Of these, 119 (48.8 %) met all eligibility criteria and were invited to participate. Of the patients who did not meet eligibility criteria (*N* = 28): 2 had severe learning disabilities and could not provide informed consent; 6 patients were too disabled to answer independently; and 10 patients did not speak enough English and did not have a translator present. Nine eligible patients (7.6 %) were unable to be approached due to time constraints in the clinic. Ten eligible patients (8.4 %) declined to participate. A total of 107 patients attending appointments did not have clinic data collected within the previously specified eligible timeframe (same day for psychological variables, ± 3 months for disease activity and disability measures), therefore precluding them from meaningful comparison with the SF-36 data (as SF-36 data would not represent current mood state, or recent disease status). In total, 100 patients were successfully recruited, yielding a total participation rate of 84.0 % (Fig. 1).

Table 1 shows the mean scores of pertinent available mental and physical variables across all patients attending appointments during the recruitment period. In comparison to patients who were recruited, ineligible patients were significantly older (*p* = 0.007), and had higher levels of pain (*p* = 0.03), disability (*p* = 0.01), and DAS28 (*p* = 0.02). Neither depression nor anxiety levels significantly differed across participation statuses.

**Table 1:** Mean scores for mental and physical health variables across attending patients

	Recruited (*N* = 100)	Not recruited (*N* = 19)	No clinic data within time-frame (*N* = 107)	Ineligible (*N* = 18)
	M (SD)	M (SD)	M (SD)	M (SD)
Age	**55.5 (15.9)**	53.8 (16.8)	55.9 (15.4)	66. 4 (14.3)**
Depression (0–6)	2.2 (2.0)	2.4 (2.0)	2.1 (1.7)	2.0 (1.2)
Anxiety (0–6)	2.0 (1.8)	1.8 (1.7)	1.7 (1.8)	2.3 (2.4)
Pain (0–100)	50.5 (25.9)*	58.3 (19.0)	49.2 (26.3)	70.4 (21.3)*
Fatigue (0–100)	54.4 (25.8)	59.9 (16.6)	50.0 (24.0)	67.0 (18.7)
HAQ	1.3 (0.8)*	1.7 (0.6)	1.2 (0.8)	2.2 (0.7)*
DAS28	3.9 (1.7)*	3.4 (1.3)	3.3 (1.6)	4.9 (1.4)*

*p < 0.5, **p < 0.01. Bold text denotes significantly different scores when compared to the recruited group. HAQ health assessment questionnaire. DAS28 28-joint disease assessment schedule

### Description of sample

Table 2 reports the descriptive statistics for the study sample. A total of 27 % of patients screened positive for pMDD and 22.5 % for pGAD. The mean disease duration was 6.5 years, and the mean DAS28 was 3.9 (SD = 1.7). On a scale of 0–100, where higher scores represent better quality-of-life, the mean MH and MCS scores were 59.7 (SD = 23.4) and 41.7 (SD = 12.5) respectively. Overall, the sample had moderate disease activity (DAS-28 m = 3.9, SD = 1.7).

**Table 2:** Description of study sample

Variable	Category/Range	N	%	M	SD
Total Sample		100			
Age	24–87			55.5	15.9
Female gender		81	81.0 %		
Ethnicity	White British	44	44.0 %		
	Asian	10	10.0 %		
	Black	20	20.0 %		
	White Other	14	14.0 %		
	Other	12	12.0 %		
Mental Health					
Depression Total	0–6			2.2	2.0
Depression Category	No symptoms	57	57.0 %		
	Some symptoms	16	16.0 %		
	pMDD	27	27.0 %		
Anxiety Total	0–6			2.0	1.8
Anxiety Category	No symptoms	63	64.3 %		
	Some symptoms	13	13.3 %		
	pGAD	22	22.5 %		
Psychological Distress	pGAD or pMDD	34	34.0 %		
Physical Health					
Disease duration (years)	0.8–34.9			6.5	6.4
Fatigue	0–100			54.4	25.8
Pain	0–100			50.5	25.9
HAQ	0–3			1.3	0.8
TJC	0–28			5.2	6.4
SJC	0–16			2.0	2.8
ESR	1–121			24.1	23.6
PGA	1–100			49.2	28.2
DAS28	0.1–7.5			3.9	1.7
SF-36 (normed subscale scores shown in brackets)					
Physical Function (PF)	0–100 (13.1–56.8)			42.3 (31.6)	28.9 (12.6)
Role Physical (RP)	0–100 (26.0–55.6)			28.6 (34.4)	39.5 (11.7)
Bodily Pain (BP)	0–90 (18.0–56.2)			36.5 (33.5)	24.1 (10.2)
Global Health (GH)	0–100 (14.2–63.8)			38.3 (33.2)	21.5 (10.7)
Vitality (VI)	0–85 (20.7–61.5)			36.5 (38.2)	23.2 (11.1)
Role Emotional (RE)	0–100 (25.4–55.7)			35.7 (36.2)	42.5 (12.9)
Social Functioning (SF)	0–100 (12.6–57.3)			55.7 (37.5)	30.6 (13.7)
Mental Health (MH)	0–100 (8.4–64.0)			59.7 (41.6)	23.4 (13.0)
PCS	13.7–55.0			31.7	9.7
MCS	18.8–69.0			41.7	12.5

*pMDD* probable major depressive disorder, *pGAD* probable generalised anxiety disorder, *HAQ* health assessment questionnaire, *TJC* tender joint count, *SJC* swollen joint count, *ESR* erythrocyte sedimentation rate, *PGA* patient global assessment, *DAS-*28 28-joint disease activity scale, *PCS SF*-36 physical component summary, *MCS* mental component summary

### Associations between variables

Table 3 summarises the correlational relationships between continuous variables.

**Table 3:** Pearson’s correlation analysis of bivariate associations

	Depression	Anxiety	MH	MCS
Depression	–			
Anxiety	0.59***	–		
MH	−0.71***	−0.70***	–	
MCS	−0.66***	−0.63***	0.91***	–
Age	−0.07	−0.23*	−0.29**	−0.32**
Disease duration	0.04	0.04	0.07	0.06
Fatigue	0.35***	0.32**	0.39***	0.39***
Pain	0.38***	0.31**	0.36***	0.30**
HAQ	0.57***	0.35**	0.46***	0.37**
TJC	0.27*	0.23*	0.29**	0.31**
SJC	0.01	0.05	0.01	0.08
ESR	0.28**	0.10	0.17	0.18
PGA	0.26*	0.27*	0.27*	0.25*
DAS28	0.35**	0.31**	0.32**	0.31**
SF-36				
PF	−0.48***	−0.36**	0.41***	0.34**
RP	−0.48***	−0.30**	0.46***	0.51***
BP	−0.47***	−0.31**	0.39***	0.41***
GH	−0.34***	−0.48***	0.58***	0.60***
VI	−0.49***	−0.40***	0.60***	0.72***
RE	−0.49***	−0.42***	0.63***	0.82***
SF	−0.63***	−0.48***	0.58***	0.67***
PCS	−0.42***	−0.28**	0.25*	0.24*

*MH SF*-36 mental health subscale, *MCS SF*-36 mental component summary, *HAQ* health assessment questionnaire, *TJC* Tender Joint count, *SJC* swollen joint count, *ESR* erythrocyte sedimentation rate, *PGA* patient global assessment, *DAS28* 28-joint disease activity score, *CRP C*-reactive protein, *BMI* body mass index, *SF-36* short form 36, *PF* physical function, *RP* role physical, *BP* bodily pain, *GH* global health, *VI* vitality, *RE* role emotional, *SF* social functioning, *PCS SF*-36 physical component summary. Depression measured via PHQ2 (score range 0–6). Anxiety measured via GAD2 (score range 0–6)

*p < 0.05. **p < 0.01. ***p < 0.001

There were several commonalities in associations between depression, anxiety MH and MCS. All three showed similar strength and direction of (or lack of) association with fatigue, pain, disability (HAQ), TJC, SJC, PGA, DAS28, and all SF-36 variables.

However whereas lower age was associated with increased anxiety, MH and MCS, no association was found between age and depression. Higher depression scores were found to be associated with increased ESR, no such association was found between ESR and anxiety MH and MCS.

### Sensitivity, specificity and receiver operating characteristic (ROC) curves for probable Major Depressive Disorder (pMDD)

**Fig. 2:**
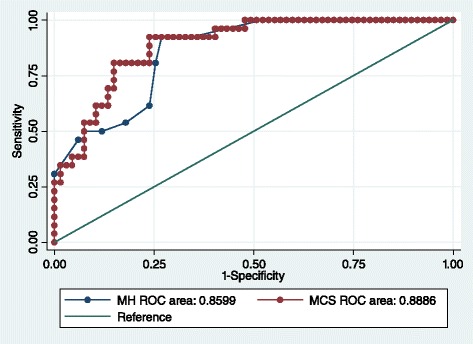
ROC for pMDD with MH and MCS SF-36 scores

The results of the ROC curve are shown in Fig. 2. The overall accuracy with which the SF-36 MH and MCS scales identify patients with pMDD are 86.0 and 88.9 % respectively. A full list of cut-points identified for pMDD using the MH and MCS are provided in the Additional file 1: Table S1.

#### SF-36 Mental Health (MH) subscale

A threshold of ≤56 provides a sensitivity of 92.6 % and a specificity of 73.2 %, correctly classifying 78.6 % of RA patients with pMDD. This is the equivalent to a normed MH subscale score of ≤40. The positive likelihood ratio of 3.5 suggests a small but important increase in the likelihood of the presence of pMDD in the case of an overall score ≤56. The negative likelihood ratio of 0.1 indicates a moderate decrease in the likelihood of pMDD in the case of an overall score of >56.

#### SF-36 Mental Component Summary (MCS)

A cut-point of 40 on the normed MCS provides a sensitivity of 92.3 % and a specificity of 70.2 %. This threshold correctly classified 76.3 % of RA patients with pMDD. A positive likelihood ratio of 3.1 suggests a small but important increase in the likelihood of pMDD being present, and the negative likelihood ratio of 0.1 suggests a moderate decrease in the likelihood of pMDD in the case of an MCS score of >40.

### Sensitivity, specificity and receiver operating characteristic (ROC) curves for probable Generalised Anxiety Disorder (pGAD)

**Fig. 3:**
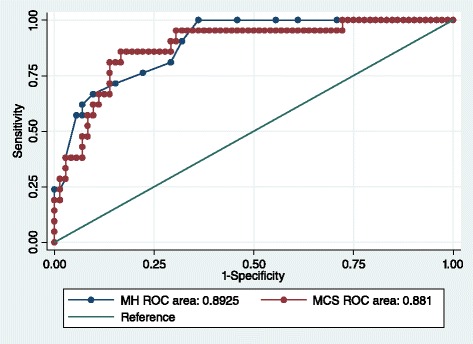
ROC for pGAD with MH and MCS SF-36 scores

The results of the ROC curve are shown in Fig. 3. The overall accuracy with which the SF-36 MH and MCS scales identify patients with pMDD are 89.3 and 88.1 % respectively. A full list of cut-points identified for pGAD using the MH and MCS are provided in the Additional file 1: Table S1.

#### SF-36 Mental Health (MH) subscale

A threshold of ≤52 provides a sensitivity of 81.0 % and a specificity of 71.4 %, correctly classifying 73.5 % of RA patients with pGAD. This is the equivalent to a normed MH subscale score of ≤37. The positive likelihood ratio of 2.8 suggests a small but important increase in the likelihood of the presence of pGAD in the case of an overall score ≤52. The negative likelihood ratio of 0.3 indicates a small but important decrease in the likelihood of pMDD in the case of an overall score of >52.

#### SF-36 Mental Component Summary (MCS)

A cut-point of 35 on the normed MCS provides a sensitivity of 85.7 % and a specificity of 81.9 %. This threshold correctly classified 83 % of RA patients with pGAD. A positive likelihood ratio of 4.7 suggests a small increase in the likelihood of pGAD being present, and the negative likelihood ratio of 0.2 suggests a moderate decrease in the likelihood of pGAD in the case of an MCS score of >35.

### Sensitivity, specificity and receiver operating characteristic (ROC) curves for any mental disorder (pMDD or pGAD)

**Fig. 4:**
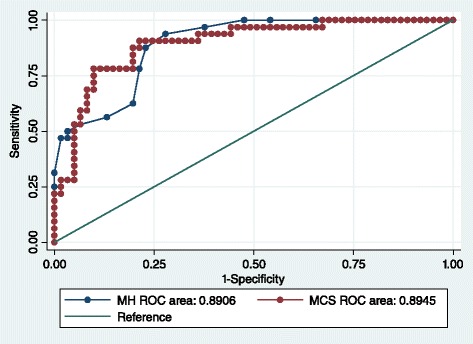
ROC for pMDD or pGAD with MH and MCS SF-36 scores

The results of the ROC curve are shown in Fig. 4. The overall accuracy with which the SF-36 MH and MCS scales identify patients with any mental disorder (pMDD or pGAD) are 89.4 % and 89.5 % respectively. A full list of cut-points identified for pMDD or pGAD using the MH and MCS are provided in the Additional file 1: Table S1.

#### SF-36 Mental Health (MH) subscale

A threshold of ≤56 provides a sensitivity of 87.9 % and a specificity of 76.9 %, correctly classifying 80.6 % of RA patients with pMDD or pGAD. This is the equivalent to a normed MH subscale score of ≤40. The positive likelihood ratio of 3.8 suggests a small but important increase in the likelihood of the presence of mental disorder in the case of an overall score ≤56. The negative likelihood ratio of 0.2 indicates a moderate decrease in the likelihood of any mental disorder in the case of an overall score of >56.

#### SF-36 Mental Component Summary (MCS)

A cut-point of 38 on the normed MCS provides a sensitivity of 87.9 % and a specificity of 76.9 %. This threshold correctly classified 82.8 % of RA patients with any mental disorder. A positive likelihood ratio of 4.4 suggests a small but important increase in the likelihood of pMDD or pGAD being present, and the negative likelihood ratio of 0.2 suggests a moderate decrease in the likelihood of pMDD or pGAD in the case of an MCS score of >38.

## Discussion

The results of this analysis suggest that the SF-36 can be used to determine the presence of pMDD, pGAD, or general psychological disorder, and potential thresholds have been suggested for these diagnoses. However optimal use of the SF-36 for screening for mental disorder may be by utilising a threshold of ≤38 on the MCS, to identify the presence of any psychological disorder. This threshold had good sensitivity (88 %) and specificity (80 %), and correctly classified 83 % of patients with either pMDD or pGAD. This can be compared to sensitivity and specificity of 95 and 66 % respectively for the NICE-recommended questions for identifying depression in patients with chronic physical health problems [13].

A similar validation study in an elderly population identified 42 as an appropriate MCS threshold to identify significant psychological distress [12]. Our identified threshold of 38 is comparable to this. As the MCS is normed, with a score of 50 (standard deviation = 10) representing the UK population, this threshold identifies patients with a score of 1 standard deviation below the population mean (scoring in the bottom 16 % of the population) as being at risk of pMDD or pGAD.

There are several elements to take into consideration when evaluating the research process described. The primary limitation is the lack of a “gold-standard” depression measure with which to validate the SF-36 domains. Employing a psychologist or psychiatrist to diagnose the presence of depression was beyond the financial and time restrictions present. We therefore relied on the measurement already taken as part of routine clinical practice, which were the PHQ9 and GAD7. Given their sensitivity and specificity of these measures against gold standard diagnostic criteria, we can expect an increase in detection of false positives. For example, if the true prevalence of depression is 20 %, and assuming an accuracy of 89 % for the PHQ9 [14] and GAD [15], our detected MCS accuracy for pMDD of 83 % would translate into a lower bound for the accuracy of 74 % for detecting true MDD. This assumed the two screening tools are independent, so it is almost certain that true accuracy is between 74 and 83 %. Although the PHQ9 and GAD7 are well-validated themselves [14, 15] future research is required to replicate the findings of this study using a gold-standard psychiatric interview against which to validate the MCS and MH scales of the SF-36. However this research has increased the utility of this widely used questionnaire and provides interesting scope for further validation and secondary analyses of existing datasets.

An additional consideration is the representativeness of our sample. As described in our methods section, patients who were unable to give consent due to learning disabilities or dementia, or who were too disabled to answer independently, we not considered eligible for inclusion in this study. This selection bias resulted in ineligible patients being significantly older, having higher levels of pain, disability and disease activity, than patients who were recruited for participation. The generalisability of our results to the wider RA population is questionable, although it is important to note that there were no differences in mental health across those who participated and those who did not.

## Conclusion

Despite these shortcomings, this analysis provides a useful first step in adding extra utility to a measurement tool already frequently used for research purposes. The addition of regular HRQoL measurement to routine RA clinical care would be beneficial, as QoL is a key outcome of importance to patients [21], and doing so would also support the need for embedded mental health care within rheumatological management practice.

## Additional file

Additional file 1: Table S1. Title of data – SF36 Mental Health (MH) and Mental Component Summary (MCS) scores as predictors of pMDD, pGAD or any psycholgical disorder (pMDD or pGAD) according to PHQ9 or GAD7 criteria. Description of data – summary of all potential thresholds identified for indicating presence of pMDD or pGAD, along with relevant sensitivity and specificity data. (DOCX 17 kb)

## Abbreviations

AUC: area under the curve
BP: SF-36 bodily pain
CI: confidence interval
DAS-28: 28-joint disease activity scale
ESR: erythrocyte sedimentation rate
GAD7: 7-item generalised anxiety disorder questionnaire
GH: SF-36 global health
HAQ: health assessment questionnaire
MCS: SF-36 mental component summary
MH: SF-36 mental health subscale
PF: SF-36 physical function
PGA: patient global assessment
pGAD: probable generalised anxiety disorder
PHQ9: 9-item patient health questionnaire
pMDD: probable major depressive disorder
RA: rheumatoid arthritis
RE: SF-36 role emotional
ROC: receiver operating characteristic
RP: SF-36 role physical
SD: standard deviation
SF: SF-36 social function
SF-36: short-form health survey
SJC: swollen joint count
TJC: tender joint count
VI: vitality
