# Supplementary material for: Usefulness of the SF-36 Health Survey in screening for depressive and anxiety disorders in rheumatoid arthritis
Source: BMC Musculoskelet Disord. 2016 May 23;17:224. doi: 10.1186/s12891-016-1083-y (PMC4878044; doi:10.1186/s12891-016-1083-y)
Supplement: Additional file 1: Table S1. — Title of data – SF36 Mental Health (MH) and Mental Component Summary (MCS) scores as predictors of pMDD, pGAD or any psycholgical disorder (pMDD or pGAD) according to PHQ9 or GAD7 criteria. Description of data – summary of all potential thresholds identified for indicating presence of pMDD or pGAD, along with relevant sensitivity and specificity data. (DOCX 17 kb) [file 12891_2016_1083_MOESM1_ESM.docx]

Table S1: SF-36 Mental Health (MH) and Mental Component Summary (MCS) scores as predictors of pMDD, pGAD or any psycholgical disorder (pMDD or pGAD) according to PHQ9 or GAD7 criteria

|  | **pMDD*** | | | | |  | **pGAD**** | | | | |  | **pMDD or pGAD***** | | | | |
| --- | --- | --- | --- | --- | --- | --- | --- | --- | --- | --- | --- | --- | --- | --- | --- | --- | --- |
|  | **Sensitivity (%)** | **Specificity (%)** | **Correctly Classified (%)** | **LR+** | **LR-** |  | **Sensitivity (%)** | **Specificity (%)** | **Correctly Classified (%)** | **LR+** | **LR-** |  | **Sensitivity (%)** | **Specificity (%)** | **Correctly Classified (%)** | **LR+** | **LR-** |
| ***MH cut-off scores*** | | | | | |  |  |  |  |  |  |  |  |  |  |  |  |
| 68 | 100.0 | 47.9 | 62.2 | 1.9 | 0.0 |  | 100.0 | 44.2 | 56.1 | 7.8 | 0.0 |  | 100.0 | 52.3 | 68.4 | 2.1 | 0.0 |
| 64 | 96.3 | 56.3 | 67.4 | 2.2 | 0.1 |  | 100.0 | 53.3 | 63.3 | 2.1 | 0.0 |  | 97.0 | 61.5 | 73.5 | 2.5 | 0.1 |
| 60 | 92.6 | 66.2 | 73.5 | 2.7 | 0.1 |  | 100.0 | 63.6 | 71.4 | 2.8 | 0.0 |  | 93.9 | 72.3 | 79.6 | 3.4 | 0.1 |
| 56 | **92.6** | **73.2** | **78.6** | **3.5** | **0.1** |  | 90.5 | 67.5 | 72.5 | 2.8 | 0.1 |  | **87.9** | **76.9** | **80.6** | **3.8** | **0.2** |
| 52 | 81.5 | 76.1 | 77.6 | 3.4 | 0.2 |  | **81.0** | **71.4** | **73.5** | **2.8** | **0.3** |  | 78.8 | 80.0 | 79.6 | 3.9 | 0.3 |
| 48 | 63.0 | 77.5 | 73.5 | 2.8 | 0.5 |  | 76.2 | 77.9 | 77.6 | 3.5 | 0.3 |  | 63.6 | 81.5 | 75.5 | 3.4 | 0.1 |
| 44 | 55.6 | 83.1 | 75.5 | 3.3 | 0.5 |  | 71.4 | 84.4 | 81.6 | 4.6 | 0.3 |  | 57.6 | 87.7 | 77.6 | 4.7 | 0.5 |
| 40 | 48.2 | 88.7 | 77.6 | 4.3 | 0.6 |  | 66.7 | 90.9 | 85.7 | 7.3 | 0.4 |  | 51.5 | 93.9 | 79.6 | 8.4 | 0.5 |
| 36 | 48.2 | 93.0 | 80.6 | 6.8 | 0.6 |  | 61.9 | 93.5 | 86.7 | 9.5 | 0.4 |  | 48.5 | 96.9 | 80.6 | 15.8 | 0.5 |
| 35 | 44.4 | 93.0 | 79.6 | 6.3 | 0.6 |  | 57.1 | 93.5 | 85.7 | 8.8 | 0.5 |  | 45.5 | 96.9 | 79.6 | 14.8 | 0.6 |
| 32 | 44.4 | 94.4 | 80.6 | 7.9 | 0.6 |  | 57.1 | 94.8 | 86.7 | 11.0 | 0.5 |  | 45.5 | 98.5 | 80.6 | 29.5 | 0.6 |
| 28 | 33.3 | 98.6 | 80.6 | 23.7 | 0.7 |  | 38.1 | 97.4 | 84.7 | 14.7 | 0.6 |  | 30.3 | 100.0 | 76.5 | - | 0.7 |
| 24 | 29.6 | 100.0 | 80.6 | - | 0.7 |  | 28.6 | 97.4 | 82.7 | 11.0 | 0.7 |  | 24.2 | 100.0 | 74.5 | - | 0.8 |
| 20 | 22.2 | 100.0 | 78.6 | - | 0.8 |  | 23.8 | 98.7 | 82.7 | 18.0 | 0.8 |  | 18.2 | 100.0 | 72.5 | - | 0.9 |
| ***MCS cut-off scores*** | | | | | |  |  |  |  |  |  |  |  |  |  |  |  |
| 70 | 100.0 | 0.0 | 28.0 | 1.0 | 0.0 |  | 100.0 | 0.0 | 22.6 | 1.0 | 0.0 |  | 100.0 | 0.0 | 34.4 | 1.0 | 0.0 |
| 65 | 100.0 | 3.0 | 30.1 | 1.0 | 0.0 |  | 100.0 | 2.8 | 24.7 | 1.0 | 0.0 |  | 100.0 | 3.3 | 36.6 | 1.0 | 0.0 |
| 60 | 100.0 | 13.4 | 37.6 | 1.2 | 0.0 |  | 100.0 | 12.5 | 32.3 | 1.1 | 0.0 |  | 100.0 | 14.8 | 44.1 | 1.2 | 0.0 |
| 55 | 100.0 | 26.9 | 47.3 | 1.4 | 0.0 |  | 100.0 | 25.0 | 41.9 | 1.3 | 0.0 |  | 100.0 | 27.9 | 52.7 | 1.4 | 0.0 |
| 50 | 100.0 | 37.3 | 54.8 | 1.6 | 0.0 |  | 95.2 | 33.3 | 47.3 | 1.4 | 0.1 |  | 96.9 | 37.7 | 58.1 | 1.6 | 0.1 |
| 45 | 100.0 | 52.2 | 65.6 | 2.1 | 0.0 |  | 95..2 | 47.2 | 58.1 | 1.8 | 0.1 |  | 96.9 | 55.8 | 69.9 | 2.2 | 0.1 |
| 40 | **92.3** | **70.2** | **76.3** | **3.1** | **0.1** |  | 95.2 | 66.7 | 73.1 | 2.9 | 0.1 |  | 90.6 | 75.4 | 80.7 | 3.7 | 0.1 |
| 38 | - | - | - | - | - |  | - | - | - | - | - |  | **87.5** | **80.3** | **82.8** | **4.4** | **0.2** |
| 35 | 80.8 | 85.1 | 83.9 | 5.4 | 0.2 |  | **85.7** | **81.9** | **82.8** | **4.7** | **0.2** |  | 78.1 | 90.2 | 86.0 | 7.9 | 0.2 |
| 30 | 53.9 | 92.5 | 81.7 | 7.2 | 0.5 |  | 57.1 | 90.3 | 82.8 | 5.9 | 0.5 |  | 53.1 | 95.1 | 80.7 | 10.8 | 0.5 |
| 25 | 30.8 | 98.5 | 79.6 | 20.6 | 0.7 |  | 33.3 | 97.2 | 82.8 | 12.0 | 0.7 |  | 25.0 | 98.4 | 73.1 | 15.3 | 0.8 |

Bold text denotes recommended cut score. MH SF-36 mental health subscale. MCS SF-36 mental component summary score. LR+ positive likelihood. LR- negative likelihood ratio. * total sample of participants used in analysis = 98. ** total sample of participants used in analysis = 93. *** total sample of participants used in analysis = 93
